# Kanglexin protects against cardiac fibrosis and dysfunction in mice by TGF-β1/ERK1/2 noncanonical pathway

**DOI:** 10.3389/fphar.2020.572637

**Published:** 2021-01-14

**Authors:** Xue Liu, Weina Han, Na An, Na Cao, Tingting Wu, Shuang Yang, Lili Ding, Xiaoli Chen, Chao Chen, Aruhan, Yannan Zhang, Kexin Wang, Lianhuan Suo, Jian Huang, Jinhui Wang, Xin Zhao, Jiuxin Zhu, Yan Zhang, Baofeng Yang

**Affiliations:** ^1^ State-Province Key Laboratories of Biomedicine-Pharmaceutics of China, Key Laboratory of Cardiovascular Medicine Research, Ministry of Education, Department of Pharmacology, College of Pharmacy, Harbin Medical University, Harbin, China; ^2^ Department of Medicinal Chemistry and Natural Medicine Chemistry, College of Pharmacy, Harbin Medical University, Harbin, China; ^3^ Science and Technology Park, Harbin Medical University, Harbin, China

**Keywords:** cardiac fibrosis, anthraquinone, Kanglexin, Extracellular Regulated Kinase 1/2 (ERK1/2), Transforming Growth Factor β1 (TGF β1)

## Abstract

Cardiac fibrosis is a common pathological manifestation accompanied by various heart diseases, and antifibrotic therapy is an effective strategy to prevent diverse pathological processes of the cardiovascular system. We currently report the pharmacological evaluation of a novel anthraquinone compound (1,8-dihydroxy-6-methyl-9,10-anthraquinone-3-oxy ethyl succinate) named Kanglexin (KLX), as a potent cardioprotective agent with antifibrosis activity. Our results demonstrated that the administration of KLX by intragastric gavage alleviated cardiac dysfunction, hypertrophy, and fibrosis induced by transverse aortic constriction (TAC) surgical operation. Meanwhile, KLX administration relieved endothelial to mesenchymal transition of TAC mice. In TGF β1-treated primary cultured adult mouse cardiac fibroblasts (CFs) and human umbilical vein endothelial cells (HUVECs), KLX inhibited cell proliferation and collagen secretion. Also, KLX suppressed the transformation of fibroblasts to myofibroblasts in CFs. Further studies revealed that KLX-mediated cardiac protection was due to the inhibitory role of TGF-β1/ERK1/2 noncanonical pathway. In summary, our study indicates that KLX attenuated cardiac fibrosis and dysfunction of TAC mice, providing a potentially effective therapeutic strategy for heart pathological remodeling.

## Introduction

Cardiovascular disease remains one of the leading causes of morbidity and mortality, which threaten human health and life throughout the world (GBD 2015 Mortality and Causes of Death Collaborators, 2016). Cardiac fibrosis is a common pathological change accompanied by various heart diseases, which is characterized by excessive synthesis and deposition of extracellular matrix (ECM) protein including collagen Ι and collagen Ш in the cardiac interstitium, resulting in mechanical sensing change and ventricular cirrhosis (Maya and Villarreal, 2010). Many kinds of cardiac diseases including hypertension, myocardial ischemia, and arrhythmia can produce cardiac fibrosis, which leads to both systolic and diastolic dysfunction and induces a variety of adverse heart events (Janicki and Brower, 2002; Berk et al., 2007). Doctors and researchers have recognized that antifibrotic therapies can improve cardiac dysfunction of patients with heart diseases (Diez et al., 2002; Fang et al., 2017; Park et al., 2019).

Anthraquinone-containing plants, such as Rhubarb, Aloe, and Semen Cassiae, have been used as traditional herbs for thousands of years. Anthraquinones are an important class of chemical compounds with extensive pharmacological activities including antibacterial (Fosso et al., 2012), anticancer (Huang et al., 2007; Su et al., 2020), antihyperglycemia (Zhang et al., 2018), cardiovascular protective (Xiao et al., 2019; Yuan et al., 2019), and neuroprotective effects (Jackson et al., 2013). In the past decade, more and more scientists are devoted to researching and developing new anthraquinone derivatives with different biological activities (Hussain et al., 2015). Accumulating evidence has revealed that anthraquinone compounds show a beneficial effect on the therapy of tissue fibrosis. Dou et al. (2019) demonstrated that aloe emodin, a natural anthraquinone derivative, alleviated renal fibrosis by regulating PI3K/Akt/mTOR signaling pathway. Recent studies have identified that emodin, a plant-derived anthraquinone, displayed strong antifibrosis properties for pulmonary (Tian et al., 2018), liver (Liu et al., 2018), renal (Ma et al., 2018), and cardiac fibrosis (Xiao et al., 2019). Lian et al. (2017) reported that chrysophanol inhibited cardiac fibrosis and injury in high-fat diet mice by regulating the Nrf2-mediated oxidant effect. Diacerein, a semisynthetic anthraquinone derivative, ameliorated cardiac fibrosis and dysfunction by inhibiting inflammation after myocardial infarction (Torina et al., 2015).

Kanglexin (KLX) is a novel anthraquinone derivative designed and synthesized by Department of Medicinal Chemistry and Natural Medicine Chemistry of Harbin Medical University. Its chemical structure is 1,8-dihydroxy-6-methyl-9,10-anthraquinone-3-oxy ethyl succinate. The introduction of monoethyl succinate substitution was beneficial to its druggability. The cardiovascular protective effects of KLX, such as antihypertension and myocardial ischemia-related cardiomyocyte pyroptosis suppression, have been recently proved (Zhao et al., 2019; Bian et al., 2020). This study aims to explore whether KLX can alleviate cardiac fibrosis and improve cardiac dysfunction of transverse aortic constriction (TAC) mice and to elucidate the underlying mechanisms.

## Materials and Methods

### Synthesis and Identification of KLX

**FIGURE 1:**
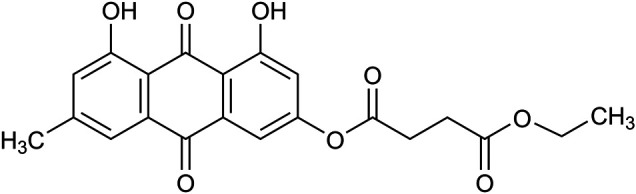
The chemical structure of KLX.

1,8-dihydroxy-6-methyl-9,10-anthraquinone-3-oxy ethyl succinate named Kanglexin (abbreviated as KLX) (Figure 1) was designed and synthesized by Department of Medicinal Chemistry and Natural Medicine Chemistry of Harbin Medical University. The synthesis and identification of KLX were performed as previously described (Li et al., 2020). The purity of KLX was over 98%, which was determined by Shimadzu LC-20A, photodiode array detector (DAD), using COSMOSIL C18 column (250 mm × 4.6 mm, 5 μm), column temperature was 40 °C, CH3OH/H2O [0.1% phosphoric acid was added in CH3OH = 88/12 (v/v)] at 1.0 mL/min, and calculating the peak areas at 254 nm. The content of KLX was preliminarily determined by HPLC area normalization method (Supplementary Figure S1).

### Animals and Animal Models

The Guideline for the Care and Use of Laboratory Animals (NIH Publication No. 82-23) was used to guide animal experiments. The Animal Ethical Committee of Harbin Medical University approved our experiment. C57BL/6J mice (aged approximately 28 weeks) were purchased from Changsheng Biotechnology Company (China). According to animals’ weight, we divided mice into 5 groups: control, TAC + vehicle, and TAC + KLX (20, 10, and 5 mg/kg/d, 0.1 ml/10 g, i.g.) groups. Animals were anesthetized with avertin (Sigma-Aldrich Corporation, United States, 0.2 g/kg, i.p.). Then, we surgically created TAC operation in mice as previously described (Yasuno et al., 2013). The sham operation was performed by open chest without aorta operation, and mice suffered to sham operation was used as the control. KLX was administered to animals for eight consecutive weeks, and other groups received an equivalent volume of solvent.

### Echocardiographic Measurements

After drug administration, mice were anesthetized with avertin (Sigma-Aldrich Corporation, United States, 0.2 g/kg, i.p.) according to their weight. After anesthesia, cardiac echocardiography was recorded by an ultrasound machine (VisualSonics Veno 2100, Canada). LVEF and LVFS were measured from M-mode recordings.

### Histological Analysis

After echocardiographic recording, 4% paraformaldehyde was used to fix heart tissues, and paraffin was used to embed the tissue and then cut the tissues into 6 μm thick slices. HE and Masson’s trichrome staining were used for the evaluation of myocardial pathological changes and collagen deposition, respectively. The tissue sections were viewed with a microscope. The cell surface area of cardiomyocytes and cardiac collagen volume fraction were measured and calculated by the Image J software.

### Measurement of Myocardial Hydroxyproline Content

For the measurement of cardiac hydroxyproline content, samples of the heart were weighted and homogenized for further detection. And then according to the instructions, a commercial hydroxyproline detection kit (Jiancheng Biotechnique Institute, China) was used to test hydroxyproline content in tissue homogenate.

### Cell Culture

Male adult C57BL/6J mice were used to isolate and culture cardiac fibroblasts (CFs). Briefly, the atrium and large blood vessels were removed from the heart. Next, we cut the ventricles into 1 mm^3^ pieces for digestion at 37°C using a digestion medium, containing 100% (w/v) trypsin and 50% (w/v) collagenase type Ⅱ for 10 min. The digestion was stopped by adding DMEM medium containing 10% FBS. The above digestion step was repeated until the tissues disappeared completely. Cells were collected by centrifuging all digestive fluid at 1,500 rpm for 5 min. Finally, we resuspended the cell pellet with a DMEM medium containing 10% FBS and cultured in a cell incubator. Cells at passage 1 were used for further experiments. After 16 h serum-free starvation, cells were stimulated by recombinant TGF-β1 (R&D Systems, United States) at the concentration of 10 ng/ml, with different concentrations of KLX (0.1, 1, and 10 μmol/L) or vehicle (0.1% dimethyl sulfoxide) for another 24 h.

Human umbilical vein endothelial cells (HUVECs) were seeded and cultured in a DMEM medium containing 10% FBS. The cells at passages 3–5 were used for subsequent study. After 12 h serum-free starvation, the cells were stimulated by recombinant TGF-β1 (R&D Systems, United States) at the concentration of 10 ng/ml, with different concentration of KLX (0.1, 1, and 10 μmol/L) or vehicle (0.1% dimethyl sulfoxide) for another 24 h.

### Methylthiazolyldiphenyl-tetrazolium bromide(MTT) Assay

Cell viability was assessed by MTT assay to measure mitochondrial succinate dehydrogenase activity in living cells. Cells were seeded in the 96-well plate and then treated with different concentrations of KLX according to the above *in vitro* methods. After drug administration, cells were incubated with MTT (0.5 mg/ml) for 4 h. After removing the media, DMSO were added to wells to dissolve formazan. The absorbance was measured at 570 nm by using a microplate spectrophotometer (Tecan, Austria).

### Cell Proliferation Assay

CFs or HUVECs were cultured in the 96-well plates and then we treated cells with different drug administration according to the above *in vitro* methods. Cell proliferation was assessed by a commercially available BrdU cell proliferation assay kit (Cell Signaling, United States) following protocols.

### Quantification of Collagen Content

CFs or HUVECs were seeded in 12-well plates. After drug administration, total soluble collagen content from the cell culture medium was quantitatively analyzed by using a commercially available soluble collagen assay kit (Sircol^TM^ Soluble Collagen Assay, Biocolor, United Kingdom).

### Immunofluorescence

Immunofluorescence staining was performed to detect the expression of CD31 and vimentin in HUVECs. Briefly, the cells were cultured on confocal dishes and received the above *in vitro* treatment. After 24 h treatment, cells were fixed with 4% paraformaldehyde for 10 min and then treated with 0.4% Triton x-100 for 1 h and 1% BSA for another 1 h at the room temperature. The cells were incubated with anti-CD31 and anti-vimentin primary antibodies at 4°C overnight. After 3-time washes with PBS, the secondary antibodies conjugated with Alexa Fluor 488 and Alexa Fluor 594 were used to incubate cells for 1 h at the room temperature. Finally, the cells were incubated with Dapi staining for 3 min. The cells were viewed with a fluorescence microscope.

### Real-Time Quantitative Reverse Transcription Polymerase Chain Reaction

We cut the heart tissue into small pieces and homogenized these pieces in Trizol reagent (Invitrogen, United States) and extracted and purified the total tissue RNA from the mouse heart. SYBR Green real-time PCR was performed to quantify the gene expression of each sample. Gapdh was used for data normalization. Primers in our study were shown in Table 1.

**TABLE 1:** Real-time qRT-PCR primer sequences

Gene	Primer sequences
Collagen Ⅰ	Forward primer: 5′-CTCGTCACAGCCTTCAC-3′
	Reverse primer: 5′-AATCCAGTAGTAATCGCTCTTC-3′
Collagen Ⅲ	Forward primer: 5′-CTACACCTGCTCCTGTCATT-3′
	Reverse primer: 5′-CCACCCATTCCTCCGACT-3′
ANP	Forward primer: 5′-ACCTGCTAGACCACCTGGAG-3′
	Reverse primer: 5′-CCTTGGCTGTTATCTTCGGTACCGG-3′
BNP	Forward primer: 5′-GAGGTCACTCCTATCCTCTGG-3′
	Reverse primer: 5′-GCCATTTCCTCCGACTTTTCTC-3′
β-MHC	Forward primer: 5′-CCGAGTCCCAGGTCAACAA-3′
	Reverse primer: 5′-CTTCACGGGCACCCTTGGA-3′
Gapdh	Forward primer: 5′-ATGTGTCCGTCGTGGATCTGA-3′
	Reverse primer: 5′-TTGCTGTTGAAGTCGCAGGAG-3′

### Enzyme-Linked Immunosorbent Assay and Western Blot

Cardiac TGF-β1 protein expression was quantitatively analyzed by using a commercial ELISA kit (Mouse TGF-β1 ELISA Kit, Cusabio, China) following the manufacturer’s protocol. Total protein of cell or tissue was extracted by using RIPA lysis buffer for subsequent Western blot assay. Nuclear extracts from hearts were collected by using a commercial Nuclear Protein Extraction Kit (Solarbio, Beijing). The concentration of protein from each sample was tested by BCA detection kit (Beyotime, China). Protein was separated by electrophoresis and transferred onto PVDF membranes. After blocking with 5% dry milk in TTBS for 1 h, we incubated the membranes with the primary antibodies against CD31, VE-cadherin, vimentin, α-SMA, phosphorylated Smad2/3 (P-Samd2/3), total Smad2/3 (t-Smad2/3), Smad-4, phosphorylated ERK1/2 (P-ERK1/2), total ERK1/2 (t-ERK1/2), gapdh, β-actin, and Histone H3 and then incubated the membranes with a fluorescence-conjugated IgG secondary antibody. Primary antibodies were purchased from BBI Life Sciences (China), Abcam (United Kingdom), Boster (China), and Cell Signaling (United States). Secondary antibodies were purchased from LI-COR Bioscience (United States). Protein expression was quantitatively analyzed by evaluating the gray-value of each protein band and the gray-value was calculated by Image J software. Gapdh, β-actin, or Histone H3 was used for data normalization.

### Statistical Analysis

Student’s *t-*test and one-way ANOVA were used for statistical comparisons for two groups and multiple groups, respectively. Kaplan-Meier’s analysis with log-rank testing was used for survival analysis. Values were expressed as mean ± SEM and *p* < 0.05 was considered significant.

## Results

### Kanglexin Improves Cardiac Function in Transverse Aortic Constriction-Operated Mice

**FIGURE 2:**
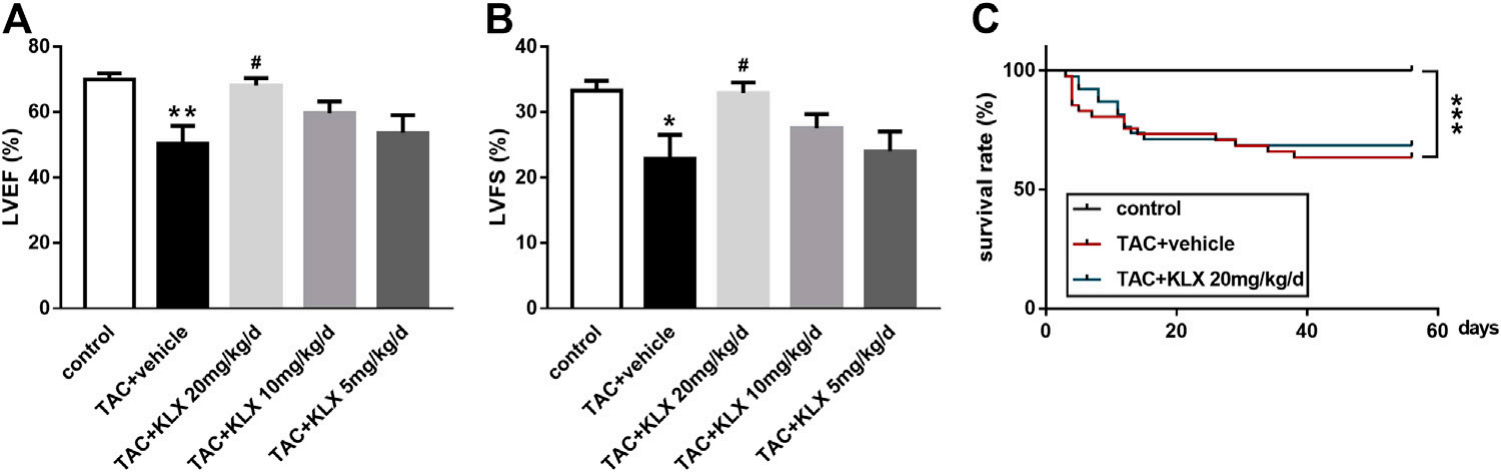
KLX alleviates cardiac dysfunction in mice of TAC. Cardiac function including LVEF% **(A)** and LVFS% **(B)** was investigated by echocardiographic analysis (*n* = 5 in each group). **(C)** Cumulative mouse mortality data of control (*n* = 35), vehicle (*n* = 41), or KLX administration at dose of 20 mg/kg/d (*n* = 38) were recorded at each time point after TAC operation. *p < 0.05, **p < 0.01, and ***p < 0.001 compared with control group and ^**#**^ *p* < 0.05 compared with TAC + vehicle group.

We used the mouse TAC model to investigate the role of this novel anthraquinone derivative (KLX) on the improvement of left ventricular dysfunction after pressure overload. The M-mode of echocardiography was performed at 8 weeks after KLX administration before scarification. Compared with the control group, mice after TAC surgery had impaired cardiac function characterized by reduced LVEF% from 69.96 ± 1.84% to 50.24 ± 5.54% and LVFS% from 33.27 ± 1.50% to 22.86 ± 3.67% (Figures 2A,B). These changes were dose-dependently alleviated by KLX at 20, 10, and 5 mg/kg/d, indicated by increased LVEF% and LVFS% compared with those of the vehicle-treated mice. LVEF% increased from 50.24 ± 5.54% to 68.16 ± 2.22%, 59.63 ± 3.61%, and 53.64 ± 5.42%, respectively (Figure 2A). LVFS% increased from 22.86 ± 3.67% to 32.88 ± 1.62%, 27.54 ± 2.15%, and 24.02 ± 3.00%, respectively (Figure 2B). Statistical analysis indicated that 20 mg/kg/d KLX treatment showed the best therapeutic effects. Thus, 20 mg/kg/d KLX was chosen to evaluate whether KLX increased the survival rate of mice after pressure overload. As illustrated in Figure 2C, the mortality rate of animals in the control group was 0%. The survival rate of mice in the TAC + vehicle group was 63.41%. However, treatment with KLX at a dose of 20 mg/kg/d showed no preventive effect on pressure overload-induced mortality and maintained the survival rate at 68.42%. These data showed that KLX improves cardiac function in TAC-operated mice, but that does not eliminate pressure overload-induced animal mortality.

### Kanglexin Prevents Cardiac Hypertrophy of Transverse Aortic Constriction Mice

**FIGURE 3:**
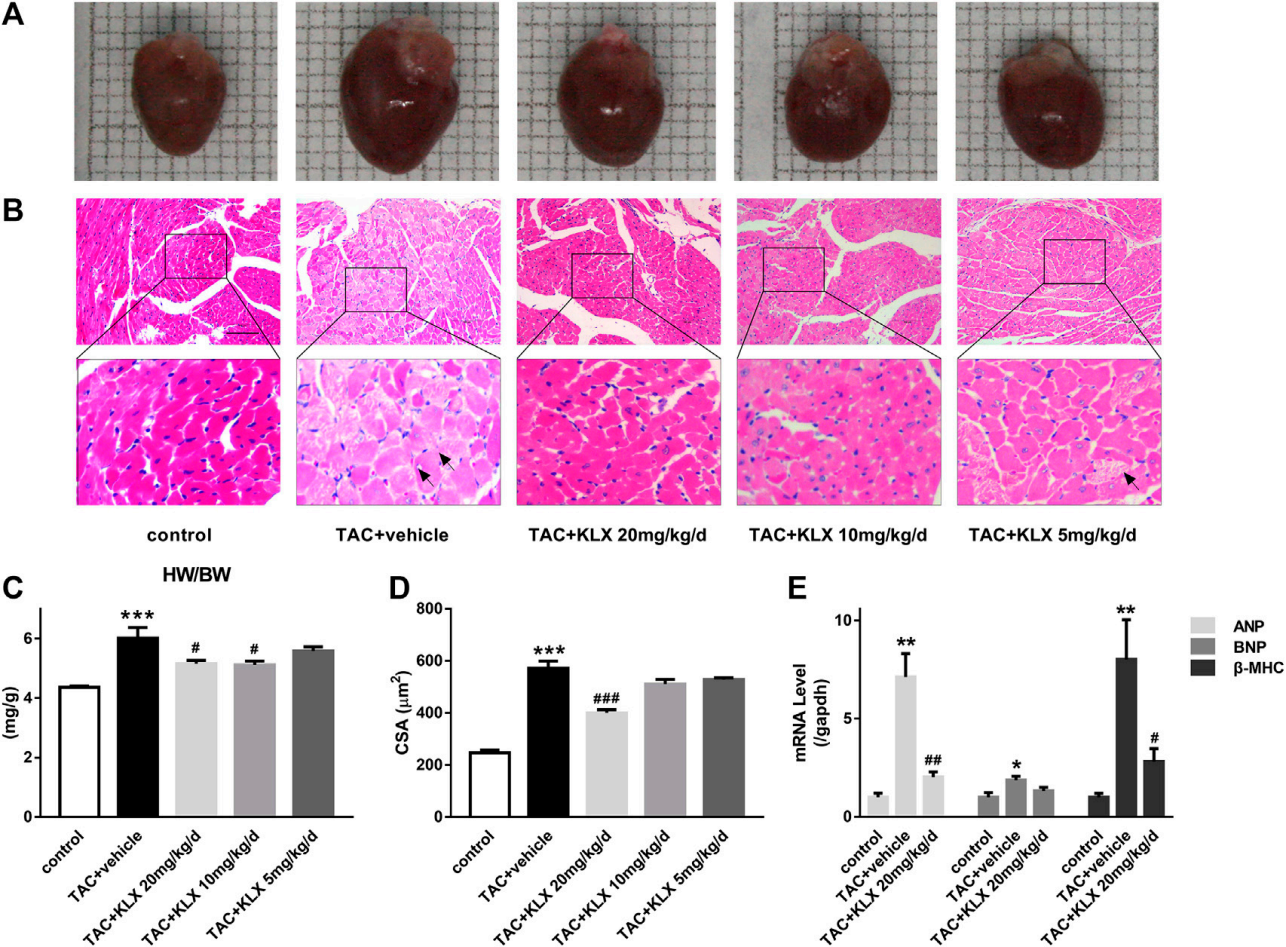
Role of KLX on TAC-induced cardiac hypertrophy. **(A)** Hearts from control, TAC + vehicle, and TAC + KLX (20, 10, and 5 mg/kg/d) groups are shown. **(B)** Heart tissues stained with HE, magnification, ×200 and scale bar = 100 µm. The black arrows point to hypertrophic cardiomyocytes. **(C)** The ratio of HW/BW revealed that KLX ameliorates cardiac index changes in TAC-operated mice (*n* = 9 in each group). **(D)** Quantitative data revealed that long-term administration of KLX dose-dependently decreased the cell surface area (CSA) during pressure overload (*n* = 5 in each group). **(E)** Pressure overload increased the transcription of ANP, BNP, and β-MHC compared with controls, which was dramatically suppressed by long-term administration of KLX (*n* = 5 in each group). *p < 0.05, **p < 0.01, and ***p < 0.001 compared with control group and ^#^ *p* < 0.05, ^##^ *p* < 0.01, and ^###^ *p* < 0.001 compared with TAC + vehicle group.

To verify whether the protective role of KLX on cardiac dysfunction was related to the inhibition of cardiac hypertrophy, the hearts were harvested, after eight-week different concentration administration. As illustrated in Figure 3A, the hearts of TAC mice were larger than those from controls, while KLX dose-dependently abolished this change. We also examined the heart weight/body weight (HW/BW) ratio of mice as a cardiac index of change in cardiac hypertrophy. The results showed that the ratio of HW/BW of TAC mice was obviously increased, indicating increased heart weight after pressure overload. In contrast, the administration of KLX prevented the increase of this hypertrophic parameter in a dose-dependent manner. HW/BW ratio increased from 6.02 ± 1.05 mg/g for vehicle cohort to 5.16 ± 0.33 mg/g, 5.11 ± 0.39 mg/g, and 5.58 ± 0.42 mg/g, respectively, at 20, 10, and 5 mg/kg/d KLX administration (Figure 3C). Additionally, we also examined the ratio of HW/BW in angiotensin Ⅱ (Ang Ⅱ) infusion mice. As expected, KLX administration mice showed dose-dependent suppression of cardiac index increase induced by Ang Ⅱ infusion (Supplementary Figure S2). Morphological analysis with HE staining confirmed that the cell surface area (CSA) of cardiomyocytes of TAC mice obviously enlarged, which was dose-dependently suppressed by KLX intragastric administration (Figures 3B,D). Furthermore, HE staining revealed that KLX prevented pathological changes in the myocardium of TAC mice, including myocardial structure disorganization, cardiomyocyte necrosis, and myofibrillar rupture (Figure 3B). At the molecular level, treatment with KLX at a dose of 20 mg/kg/d downregulated the transcription levels of hypertrophic markers of TAC mice, including ANP, BNP, and β-MHC (Figure 3E). These data indicate that KLX exerts a preventive effect on cardiac hypertrophy.

### Kanglexin Alleviates Cardiac Fibrosis of Transverse Aortic Constriction Mice

**FIGURE 4:**
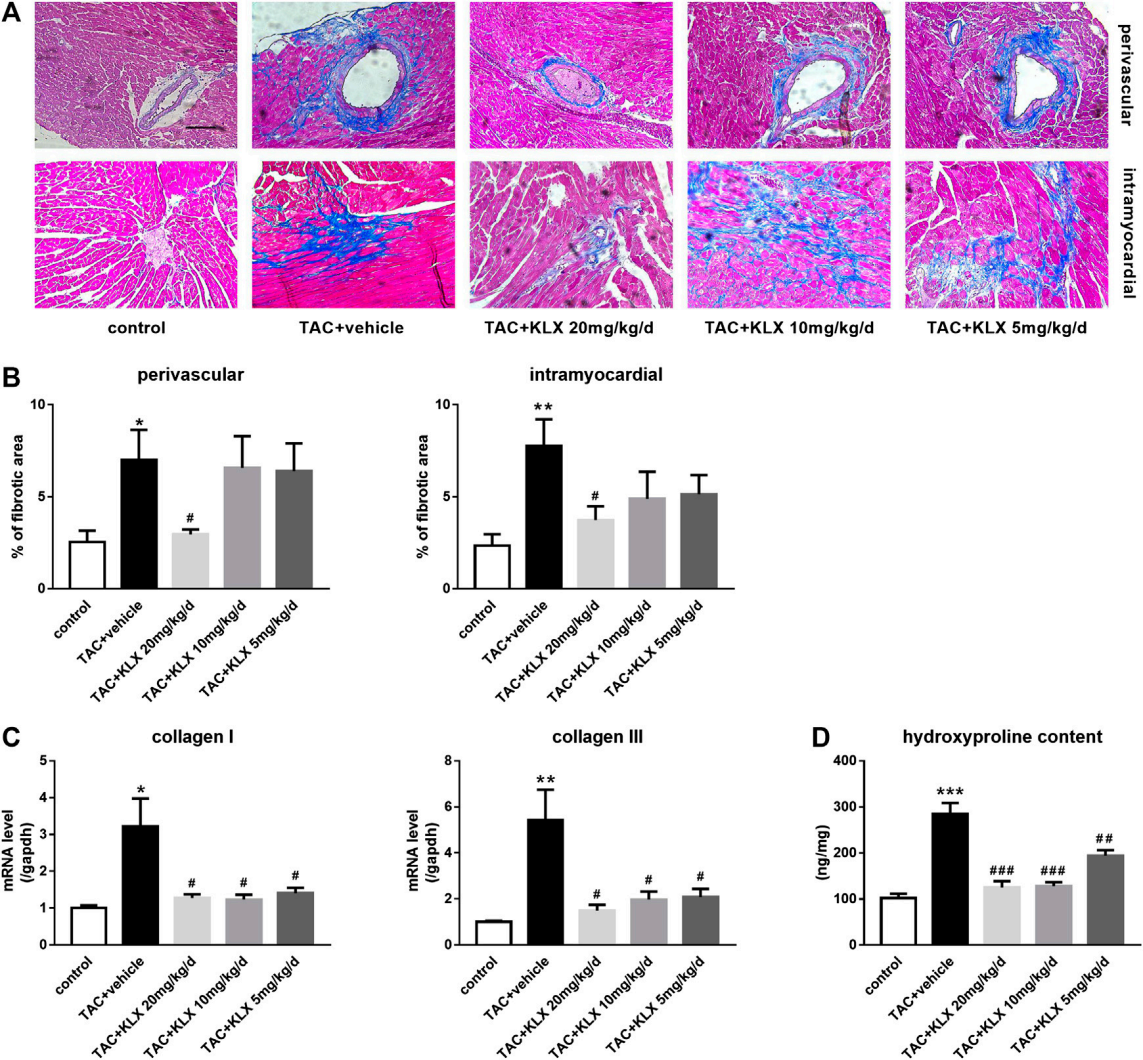
Role of KLX on cardiac fibrosis. **(A)** Heart tissues stained with Masson’s trichrome. Blue areas indicate collagen deposition, magnification, ×200 and scale bar = 100 µm. **(B)** Quantitative data showed that collagen synthesis and deposition increased markedly in TAC-operated groups compared with controls in both perivascular area and intramyocardial area; KLX administration at dose of 20 mg/kg/d strongly prevented collagen accumulation (*n* = 5 in each group). Pressure overload significantly upregulated collagen Ⅰ and collagen Ⅲ transcription (**C**; *n* = 5 in each group) and cardiac hydroxyproline content compared with controls (**D**; *n* = 9 in each group), which was significantly suppressed by long-term administration of KLX in a dose-dependent manner. *p < 0.05, **p < 0.01, and ***p < 0.001 compared with control group and ^**#**^ *p* < 0.05, ^**##**^ *p* < 0.01, and ^**###**^ *p* < 0.001 compared with TAC + vehicle group.

To verify the possible role of KLX in modulating cardiac fibrosis, we performed Masson’s trichrome staining to test collagen production and deposition in the myocardium. Our results showed that collagen synthesis and deposition increased markedly in TAC-operated groups compared with controls in both the perivascular area and the intramyocardial area (Figures 4A,B). KLX administration at a dose of 20 mg/kg/d strongly prevented collagen accumulation (Figures 4A,B). Additionally, the antifibrotic effect of KLX on the heart of TAC mice was also confirmed by dose-dependent suppression of the increased collagen Ⅰ and collagen Ⅲ transcription (Figure 4C). Consistent with the above results, the left ventricular hydroxyproline content in the TAC + vehicle group was upregulated as compared with controls, whereas the hydroxyproline content was dose-dependently downregulated by KLX intragastric administration (Figure 4D). We also examined the left ventricular hydroxyproline content in Ang Ⅱ infusion mice. As expected, the hearts in the KLX treatment group showed dose-dependent suppression of the upregulation of hydroxyproline content induced by Ang Ⅱ (Supplementary Figure S3). The above data demonstrate that KLX affects cardiac fibrosis.

### Kanglexin Relieves Fibroblast Activation and Function Induced by TGF-β1 in Primary Cultured Adult Mouse Cardiac Fibroblasts

**FIGURE 5:**
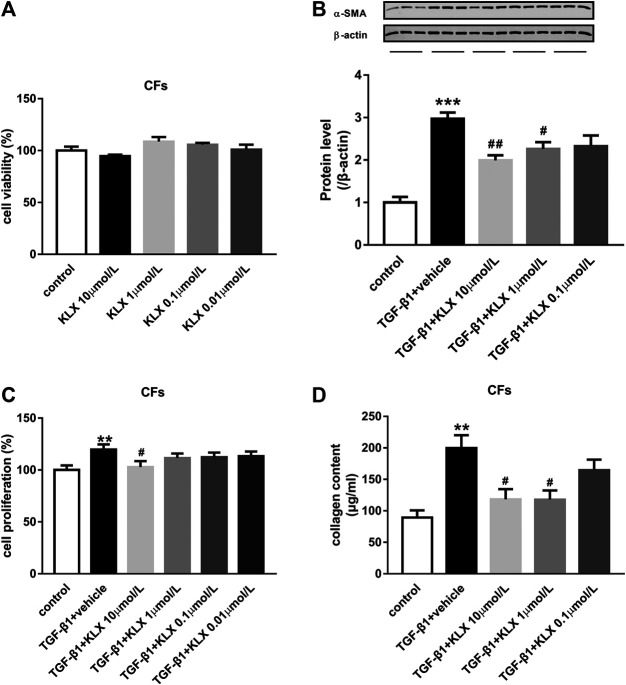
KLX relieves fibroblast activation and function induced by TGF-β1 in primary cultured adult mouse CFs. **(A)** KLX showed no significant effect on cell viability of CFs. TGF-β1 significantly upregulated α-SMA expression **(B)**, proliferation **(C)**, and collagen secretion **(D)** in adult mouse CFs compared with untreated cells, which was concentration-dependently suppressed by KLX cotreatment (*n* = 3–4 in independent experiments). **p < 0.01 and ***p < 0.001 compared with control group and ^**#**^ *p* < 0.05 and ^**##**^ *p* < 0.01 compared with TAC + vehicle group.

The activation of CFs and subsequent collagen secretion are the key procedure to drive the fibrogenesis response in case of cardiac stress. In the current study, primary cultured adult mouse CFs were used to explore the regulatory role of KLX on fibrogenesis response *in vitro*. We first examined the basic cell viability of CFs in response to different concentrations of KLX (0.01, 0.1, 1, and 10 μmol/L). After 24 h treatment, the cell viability was not apparently changed in CFs (Figure 5A). It means that KLX cannot alter the basic cell viability of CFs. TGF-β1 is an important cytokine, which has been shown to stimulate fibroblast activation (Ruiz-Ortega et al., 2007). To explore whether KLX directly affected CFs to protect the heart against fibrosis, CFs were stimulated by TGF-β1 at the concentration of 10 ng/ml for 24 h with or without a different concentration of KLX (0.1, 1, and 10 μmol/L). Because the process of fibroblasts to myofibroblasts activation, characterized by overexpression of α-SMA, plays a key role in cardiac fibrosis, we then examined the expression of α-SMA in TGF-β1-stimulated CFs with or without KLX. As expected, the expression of α-SMA in TGF-β1-stimulated CFs was significantly increased, which were concentration-dependently abolished by cotreatment with KLX (Figure 5B). However, of note is that significant effects of KLX on CFs activation were observed only at the concentration of 1 and 10 μmol/L, even though the similar change was also observed with the lowest concentration. In response to injurious stimuli, fibroblasts proliferate rapidly in the injured myocardium. We performed BrdU cell proliferation assay to demonstrate the role of KLX on TGF-β1-induced cell proliferation. Our results showed that TGF-β1 treatment stimulated fibroblast proliferation; KLX cotreatment eliminated TGF-β1-induced proliferative response in CFs (Figure 5C). The deposition of collagen leads to the remodeling of ECM in fibrous myocardium. The key role of activated fibroblasts in the pathological remodeling ECM is to secrete collagen. We detected collagen content in culture medium to examine whether KLX played a regulatory role in collagen production and secretion of CFs. Our data revealed that TGF-β1 induced a remarkable increase of collagen content in culture medium, but only higher concentration (1 and 10 μmol/L) of KLX decreased these changes (Figure 5D).

### Kanglexin Attenuates the Endothelial to Mesenchymal Transition

**FIGURE 6:**
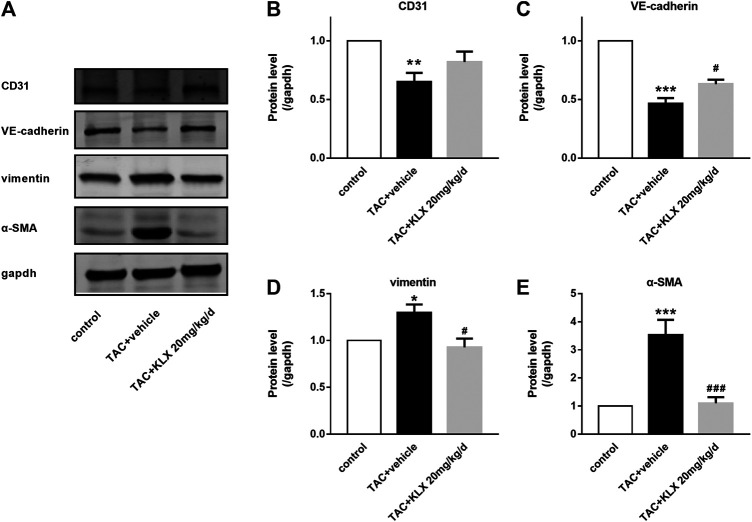
KLX alleviates EndMT in TAC-pressured heart. **(A)** Western blot analysis of CD31, VE-cadherin, vimentin, and α-SMA in mouse hearts. The relative protein levels of CD31 **(B)**, VE-cadherin **(C)**, vimentin **(D)**, and α-SMA **(E)** in mouse hearts (*n* = 4 in each group). *p < 0.05, **p < 0.01, and ***p < 0.001 compared with control group and ^**#**^ *p* < 0.05 and ^**###**^ *p* < 0.001 compared with TAC + vehicle group.

Endothelial cells are one of the main sources of myofibroblasts, which have been demonstrated and involved in cardiac fibrosis through EndMT (Sanchez-Duffhues et al., 2018). Therefore, we investigated the potential regulation of KLX on EndMT in TAC mice. Our experiments showed that KLX markedly prevented the upregulation of mesenchymal-specific markers (α-SMA and vimentin) and the downregulation of endothelial-specific markers (VE-cadherin and CD31) of the TAC mice (Figures 6A–E). These results suggest that KLX attenuates TAC-induced EndMT.

**FIGURE 7:**
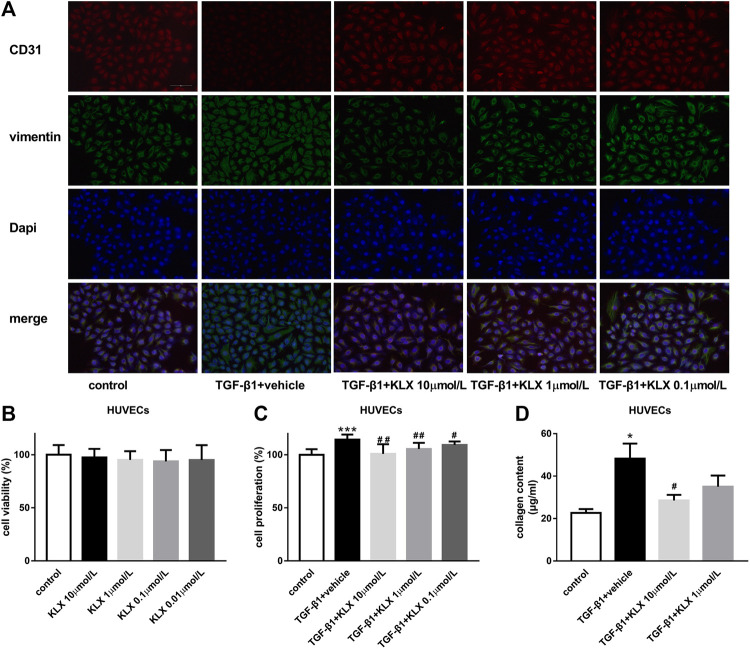
KLX suppresses EndMT stimulated by TGF-β1 in HUVECs. **(A)** Representative immunofluorescence images showing staining of endothelial marker CD31 and mesenchymal marker vimentin in different treatment of HUVECs, magnification, ×200 and scale bar = 100 µm. **(B)** KLX showed no significant effect on cell viability of HUVECs. TGF-β1 significantly increased cell proliferation **(C)** and collagen secretion **(D)** in HUVECs compared with untreated cells, which was concentration-dependently suppressed by KLX cotreatment (*n* = 3–4 in independent experiments). *p < 0.05 and ***p < 0.001 compared with control group and ^**#**^ *p* < 0.05 and ^**##**^ *p* < 0.01 compared with TAC + vehicle group.

To confirm the direct influence of KLX on endothelial cells, HUVECs were applied for our following *in vitro* experiments. We first examined the basic cell viability of HUVECs in response to different concentrations of KLX (0.01, 0.1, 1, and 10 μmol/L). KLX did not affect the cell viability of HUVECs after 24 h treatment (Figure 7B). It means that KLX cannot alter the basic cell viability of HUVECs. After heart injury, endothelial cells proliferation promotes EndMT and induces cardiac fibrosis. Therefore, we then evaluated the role of KLX on TGF-β1-induced HUVECs proliferative response. HUVECs were cultured and stimulated by TGF-β1 at the concentration of 10 ng/ml for 24 h with or without different concentration of KLX (0.1, 1, and 10 μmol/L). As shown in Figure 7C, we found a proliferative response following treatment with TGF-β1, while KLX concentration-dependently reduced the proliferation of HUVECs stimulated by TGF-β1. Collagen secretion from endothelial cells reveals evidence of EndMT. We next examined whether KLX played a role in the regulation of collagen synthesis and secretion in HUVECs. Our results revealed that TGF-β1 induced a significant increase of collagen content in culture medium, but KLX concentration-dependently reversed these changes. Of note, the statistical difference was only found in the high concentration group (10 μmol/L) (Figure 7D). Additionally, the results from double immunofluorescence staining of CD 31 and vimentin confirmed that endothelial cells with KLX pretreatment inhibited TGFβ1-induced EndMT with an increase for the staining of CD31 and decrease for the staining of vimentin (Figure 7A). These data indicate that KLX target endothelial cells to protect the heart against fibrosis.

### The Effect of Kanglexin on Canonical and Noncanonical Pathways of TGF-β1 Signaling

**FIGURE 8:**
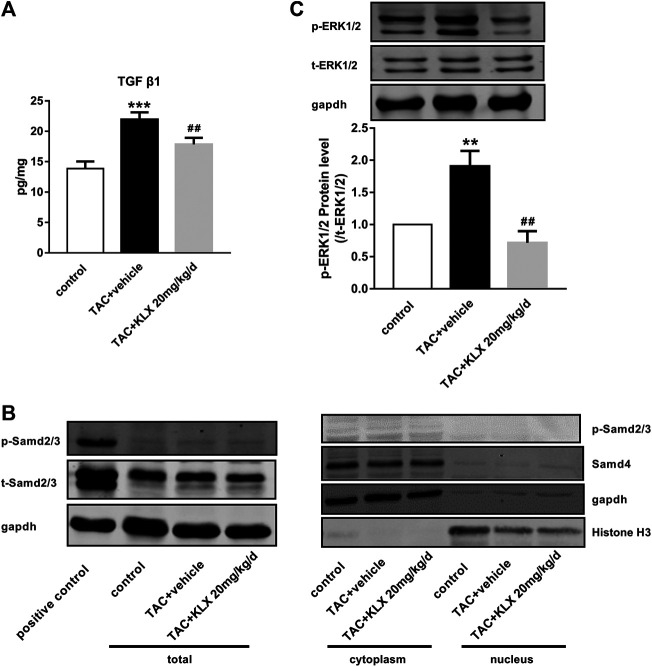
The effect of KLX on canonical and noncanonical pathways of TGF-β1 signaling. **(A)** ELISA results showed that cardiac TGF-β1 protein expression was upregulated in long-term pressure overload heart compared with the control cohort, which was reversed by KLX administration (*n* = 7 in each group). **(B)** The expression levels of p-Smad2/3, t-Smad2/3, and Smad4 in mouse hearts were measured by Western blot. TAC-operated hearts showed no significant P-Smad2/3 protein expression in total, cytoplasmic, and nuclear fractions. TAC-operated hearts exhibited no significant nuclear expression of Smad4. TGF-β1-stimulated HUVECs, as a positive control, showed a highlighted protein band of p-Samd2/3, indicating activation of Smad2/3. **(C)** The relative expression levels of p-ERK1/2 and t-ERK1/2 in mouse hearts were measured by Western blot. TAC operation significantly increased ERK phosphorylation compared with controls, which was suppressed by KLX administration (*n* = 5 in each group). **p < 0.01 and ***p < 0.001 compared with control group and ^**##**^ *p* < 0.01 compared with TAC + vehicle group.

In order to explore the molecular mechanisms through which KLX regulate cardiac fibrosis, the associated signaling pathways at the protein level were screened. TGF-β1 is a key cytokine that induces fibrogenesis response in a stress heart. Our data showed that TGF-β1 expression in the left ventricle was increased in long-term pressure overload heart compared with controls, which was reduced by KLX intragastric administration (Figure 8A). We then examined the effects of KLX on TGF-β1/Smads canonical pathway. Interestingly, TAC-operated hearts exhibited no significant phosphorylated expression level of Smad2/3 (P-Smad2/3) and nuclear expression levels of Smad4, suggesting that the heart during pressure overload had a blunted response to TGF-β1/Smads pathway (Figure 8B). In contrast, TGF-β1-stimulated HUVECs, as a positive control, showed a highlighted protein band of p-Samd2/3, indicating activation of TGF-β1/Smads pathway. In addition to the Smads pathway, TGF-β1 also induce several noncanonical pathways of TGF-β1 signaling, among which mitogen-activated protein kinase, ERK, has been proved to be involved in TGF-β1-induced fibrogenic response (Xu et al., 2017). In this study, we verified whether KLX inhibited cardiac fibrosis by modulating ERK1/2 activation. Our data showed that phosphorylated ERK1/2 (p-ERK1/2) was upregulated in TAC-operated hearts compared with controls. KLX was able to inhibit the upregulation of p-ERK1/2 expression significantly in mouse hearts after TAC operation (Figure 8C).

## Discussion

Anthraquinone compounds exhibit a series of biological activities, but their application as new drug candidates are limited by their physical and chemical properties. The druggability related parameters, including Gibbs free energy, topological Polar Surface Area (tPSA) and logP of the anthraquinone derivative (KLX) were evaluated according to ChemDraw professional software. Gibbs-free energy of KLX was reduced from −357.13 kJ/mol of the original anthraquinone compound to −782.36 kJ/mol, which will increase the binding with its target theoretically. Chemicals usually have favorable intestinal absorption, if their tPSA are lower than 140.2 (Ertl et al., 2000; Stenberg et al., 2000). By ChemDraw professional software, tPSA of KLX is 127.2, which means it has high success rate as drug candidate. The logP of KLX is also appropriately increased to 1.98. Therefore, compared with nature anthraquinone compounds, the chemical structure of KLX was beneficial to its druggability.

Cardiac fibrosis is a common pathological change accompanied by a variety of heart diseases, which increases the incidence and mortality of many cardiovascular diseases (Massare et al., 2010). It has been recognized that antifibrotic therapies are useful in improving cardiac dysfunction of patients with heart diseases (Diez et al., 2002; Fang et al., 2017; Park et al., 2019). In the present study, we focused on investigating the therapeutic effect on cardiac dysfunction and cardiac fibrosis in long-term pressure overload mouse hearts. Our results demonstrated that long-term administration of KLX improved the cardiac dysfunction of TAC mice, including decreasing heart index, the degree of cardiac hypertrophy, and reducing collagen deposition in the interstitial and perivascular space. We also found that KLX treatment downregulated the increased heart index and cardiac hydroxyproline content of experiment mice to chronic Ang Ⅱ stimulation. It suggests that KLX can alleviate cardiac structural remodeling of hypertensive mice induced by long-term persistent Ang Ⅱ stimulation (Supplementary Figures S2,S3). In addition to the present study, our previous research revealed that KLX reduced the infarct size and attenuates cardiac dysfunction of myocardial infarction mice (Bian et al., 2020). Therefore, KLX can improve cardiac structural and functional remodeling in different types of heart disease.

We next verified the regulatory role of KLX (a novel anthraquinone derivative) on fibrogenic responses in cultured CFs. Herein, we found that KLX at the concentration of 1 and 10 μmol/L prevented cardiac fibrogenesis via directly inhibiting the transformation of fibroblasts to myofibroblasts, cell proliferation, and collagen secretion induced by TGF-β1. Furthermore, more and more pieces of evidence support that endothelial cells are the main source of myofibroblasts during cardiac fibrosis (Sanchez-Duffhues et al., 2018). A landmark study published in 2007 confirmed that EndMT is involved in cardiac fibrosis of the adult heart (Zeisberg et al., 2007). Many studies demonstrated that the inhibition of EndMT might be a useful strategy in the therapy of cardiac fibrosis (Song et al., 2019; Tsai et al., 2019). In this study, we demonstrated the potential regulation of KLX on EndMT in TAC mice. Our experiments showed that KLX markedly prevented the upregulation of mesenchymal markers (vimentin and α-SMA) and the downregulation of endothelial-specific markers (CD31 and VE-cadherin) of the TAC mice, which indicates that KLX attenuates TAC-induced EndMT. Meanwhile, we found that the inhibitory role of KLX on cell proliferation and collagen secretion in TGF-β1-treated HUVECs demonstrated its direct regulatory effect on endothelial cells.

**FIGURE 9:**
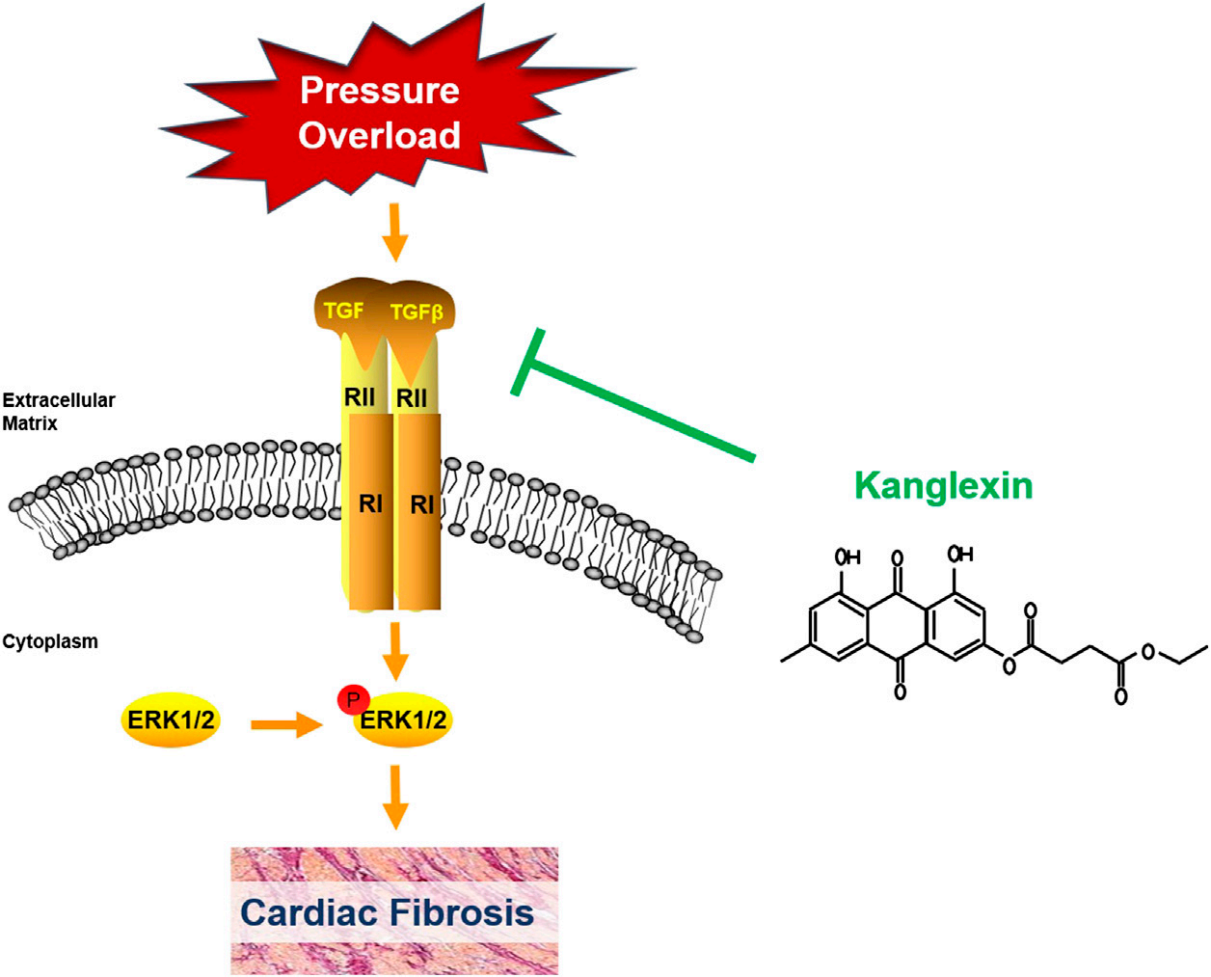
Schematic diagram of the underlying mechanisms by which KLX exerts a regulatory role in cardiac fibrosis.

Although there are many factors and regulators affecting cardiac fibrosis, TGF-β1 is an important cytokine involved in the process of cardiac fibrosis (Ruiz-Ortega et al., 2007). Our results showed that cardiac TGF-β1 expression was dramatically downregulated in KLX administration mice than vehicle cohort in response to long-term pressure overload. Corresponding to our phenotypic verification findings of *in vivo* and *in vitro* study, the results of cardiac TGF-β1 expression in TAC mice with or without KLX administration further implicated that KLX could effectively attenuate cardiac fibrosis. As an upstream regulator, TGF-β1 activates downstream canonical and noncanonical signaling cascades. In TGF-β1 canonical pathway, activated TGF-β1 binds to the transmembrane TGF-βR II, which results in the recruitment of TGF-βR Ⅰ. TGF-β1/TGF-βR II/TGF-βR Ⅰ complex subsequently triggers the phosphorylation of Smad2/3 and then phosphorylated Smad2/3 recruits Smad4. The complex of Smad2/3/4 migrates into the nucleus and regulates ECM-associated gene transcription and then stimulates fibroblast activation and collagen production (Shi and Massague, 2003; Bujak and Frangogiannis, 2007). It is reported that TGF-β1/Smads signaling pathway is apparently downregulated in mouse CFs from the elder in comparison with CFs from the young (Bujak et al., 2008). That means the sensitivity of TGF-β1/Samds canonical signaling pathway is related to age. The TAC mice in our study were approximately 36 weeks old, when sacrificed for further investigation, so the response of TGF-β1/Smads signaling of hearts was inclined to the elder animal. The blunted TGF-β1/Smads response of the hearts to long-term pressure overload stimulation was consistent with their age in our present study. In addition to the Smads pathway, TGF-β1 also induce several noncanonical pathways of TGF-β1 signaling, among which mitogen-activated protein kinase, ERK, has attracted the attention of many researchers. TGF-β1 activates TGF-βR Ⅰ and then directly phosphorylates ShcA to induce the recruitments of Grb2 and Sos complex and finally induces the ERK1/2 activation (Lee et al., 2007). Importantly, various drugs protect the heart against pathological remodeling by regulating the ERK1/2 signaling pathway (Zhang et al., 2016; Tang et al., 2018; Wang et al., 2018). Our data showed that KLX was able to inhibit the upregulation of phosphorylated ERK1/2 expression significantly in mouse hearts after TAC operation, which indicated that TGF-β1/ERK1/2 noncanonical but not TGF-β1/Smads canonical pathway was involved in the antifibrosis effect of KLX on hearts of pressure overload mice (Figure 9). These findings do not exclude the possibility of other signaling pathways by which KLX exerts a regulatory role in cardiac fibrosis, and this notion needs further investigation.

## Conclusion

Our study provides evidence that KLX (1,8-dihydroxy-6-methyl-9,10-anthraquinone-3-oxy ethyl succinate) alleviates cardiac dysfunction and fibrosis after pressure overload. Furthermore, TGF-β1/ERK1/2 noncanonical pathway attributes to the beneficial effect of KLX on cardiac fibrosis. In order to develop KLX as a cardioprotective candidate agent, pharmacokinetic studies will be carried out in the future.

## Data Availability Statement

The raw data supporting the conclusions of this article will be made available by the authors, without undue reservation.

## Ethics Statement

This study was carried out in accordance with the recommendations of US National Institutes of Health (NIH) guidelines for the care and use experimental animals. The protocol was approved by Ethics Committee of Harbin Medical University.

## Author Contributions

YZ and BfY participated in research design; NC and YnZ contributed to project administration and supervision; XL and WnH contributed to original draft preparation; NA, LlD, XlC, TtW, and SY conducted the pharmacological experiments; XZ and JxZ performed the data ananlysis; AR prepared the required compound; JH and JW designed the compound; LhS, CC, and KxW conducted supplementary experiments. All authors approved the submitted version of manuscript.

## Funding

This study was supported by the NSFC-FRQS project (2019-2021), National Natural Science Foundation of China (81730012/81870259/81700220), Heilongjiang Province Science Fund for Returnees (LC2017033), Heilongjiang Postdoctoral Fund (LBH-Z18183), and National Science and Technology Major Project of the Ministry of Science and Technology of China (2018ZX09735005).

## Conflict of Interest

The authors declare that the research was conducted in the absence of any commercial or financial relationships that could be construed as a potential conflict of interest.
