# Supplementary material for: Kanglexin protects against cardiac fibrosis and dysfunction in mice by TGF-β1/ERK1/2 noncanonical pathway
Source: Front Pharmacol. 2021 Jan 14;11:572637. doi: 10.3389/fphar.2020.572637 (PMC7840489; doi:10.3389/fphar.2020.572637)
Supplement: Supplementary file 1 [file datasheet1.docx]

Supplementary Material

## Supplementary Figures


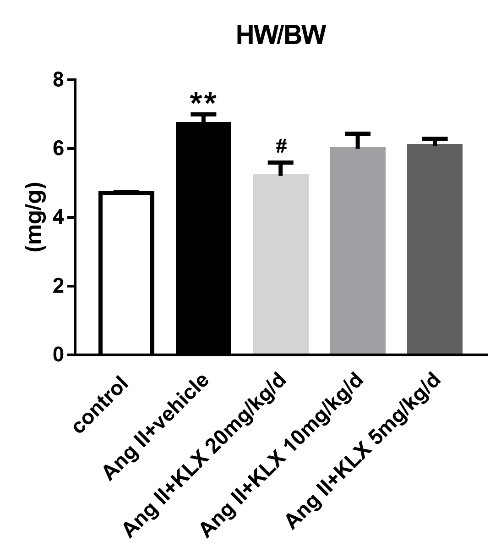


**Supplementary Figure 1.** **Effect of KLX on the ratio of HW/BW in** **Ang Ⅱ infusion mice** (n=3 in each group). ***P* < 0.01 compared with control group, **^#^***P* < 0.05 compared with Ang Ⅱ+vehicle group.

.
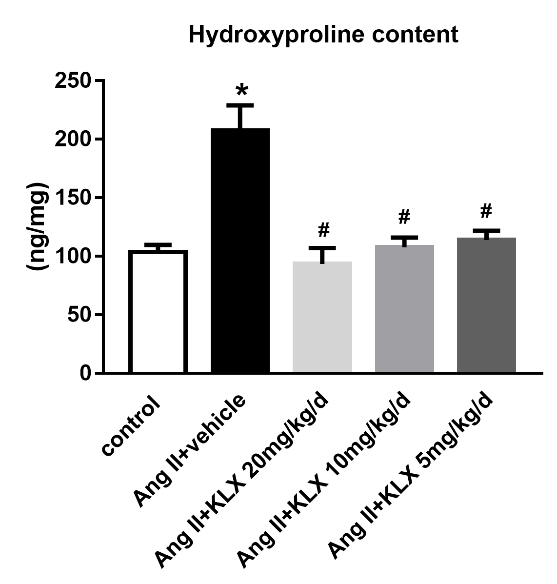


**Supplementary Figure 2. KLX decreases cardiac hydroxyproline content in Ang Ⅱ infusion mice.** (n=3 in each group). **P* < 0.05 compared with control group, **^#^***P* < 0.05 compared with Ang Ⅱ+vehicle group.

1. **Supplementary materials and methods**
   1. **Ang Ⅱ infusion animal model**

Male C57BL/6 were purchased from Changsheng Biotechnology Company (China). According to animals’ weight, we divided mice into 5 groups: control, Ang Ⅱ+vehicle, Ang Ⅱ+KLX (20, 10 and 5 mg/kg/d) groups. Animals were anaesthetized with avertine (Sigma-Aldrich Corporation, USA, 0.2g/kg, i.p.) according to their weight. Then, we created a surgical operation to mice by subcutaneous implantation with a 100 μL osmotic pump (model Alzet 1004; Durect Corporation, CA) containing a solution of angiotension Ⅱ (Ang Ⅱ) in saline. Control mice were implanted with a 100 μL osmotic pump of slow-release saline. Ang Ⅱ infusion pump infused Ang Ⅱ at 35 μg/kg/h. KLX was administered to animals daily (i.g.), and vehicle group received an equivalent volume of solvent. Hearts were collected for subsequent experiments after 4 weeks of Ang Ⅱ or saline infusion.
